# Information needs of Lynch syndrome and BRCA 1/2 mutation carriers considering risk-reducing gynecological surgery: a qualitative study of the decision-making process

**DOI:** 10.1186/s13053-024-00278-4

**Published:** 2024-05-02

**Authors:** Lucy Zhao, Lorrie Lynch, Lua Eiriksson

**Affiliations:** 1 Faculty of Health Sciences, McMaster University, Hamilton, ON Canada; 2 Cancer Genetics Clinic, Juravinski Hospital and Cancer Centre, Hamilton, Canada; 3 Division of Gynecologic Oncology, Juravinski Hospital and Cancer Centre, Hamilton, Canada; 4 Department of Obstetrics and Gynecology, McMaster University, Hamilton, Canada

**Keywords:** Lynch syndrome, BRCA, Prophylactic surgery, Decision-making

## Abstract

**Background:**

Risk-reducing gynecological surgery (RRGS) is a prophylactic procedure that may be offered to BRCA1, BRCA2, and Lynch syndrome (LS) mutation carriers to reduce the risk of developing gynecological cancer. This study was conducted to better understand patients’ information needs and evaluate how patients weigh different sources of information in their decision-making process surrounding RRGS.

**Methods:**

This study used a qualitative approach to understanding women’s perspectives towards RRGS. Semi-structured interviews were conducted virtually with 8 women. Women offered RRGS between 35 and 70 years of age who are English-speaking and have an identifiable BRCA or LS mutation were included. Data from interviews was coded with constant comparative analysis to develop themes.

**Results:**

Of the eight women, six had selected to undergo either prophylactic hysterectomy or oophorectomy: 5 decided yes to RRGS; 1 decided no; 2 were undecided. Thematic analysis found that the key factors affecting women’s decisions around prophylactic surgery were cancer risk, surgical menopause, and psychological readiness. To make an informed decision, women relied most heavily on information provided by healthcare professionals (e.g. doctors, genetic counselors) and family members with prior cancer experience. However, some women reported that they did not feel adequately informed enough to make a decision and identified COVID-19 as a significant barrier affecting access to information.

**Conclusion:**

This qualitative study revealed the key sources of information influencing attitudes regarding RRGS and how women consulted different sources of information to reach a decision. Results underscore the need for greater attention to women’s information needs in the context of psychological readiness, particularly amidst the pandemic. Research involving a larger sample size may help to better inform how support can be provided to individuals with BRCA and LS mutations considering RRGS.

## Introduction

Individuals with cancer susceptibility syndromes such as BRCA1, BRCA2, and Lynch syndrome mutations face a greater lifetime risk of developing endometrial, ovarian, fallopian tube, and peritoneal cancers. Lynch syndrome (LS) is a hereditary cancer susceptibility syndrome caused by a pathogenic germline mutation in the *EPCAM* gene or one of the DNA mismatch repair (MMR) genes *MLH1, MSH2, MSH6, PMS2* [1]. Individuals with LS face a 41–70% risk of colorectal cancer, 40–60% risk of endometrial cancer, and 10–24% ovarian cancer [2–6]. The penetrance for LS-related malignancies varies based on the individual’s biological sex and the mutation type where cancer rates are highest in individuals with MSH6 variants and lowest in those with PMS2 variants [7, 8]. On the other hand, *BRCA1* and *BRCA2* carriers face a 50–59% and 42–51% breast cancer risk, and 34–45% and 13–21% ovarian cancer risk respectively, as well as a two-to threefold risk of endometrial cancer [9].

Individuals affected by BRCA and LS mutations require early surveillance and screening to detect potential malignancies. For BRCA carriers, breast awareness is recommended every 6–12 months from the age of 18 years and clinical breast examination is recommended every 6–12 months from age 25 [10]. However, endometrial cancer screening and surveillance for BRCA carriers is currently not indicated in clinical guidelines [10]. While BRCA and LS carriers also face increased risk of ovarian cancer, the benefit of this screening is unclear [11]. For individuals with LS or their first-degree relatives, US Multi-Society Task Force on Colorectal Cancer recommends colonoscopic every 1–2 years starting between the ages of 20 and 25, or 2–5 years before the youngest age of diagnosis of colorectal cancer in the family if diagnosed before 25 years of age [12]. Consideration may be given to started screening later at age 30 for those with MSH6 mutations and at age 35 in PMS2 [12, 13]. Endometrial sampling and transvaginal ultrasonography of the uterus is recommended beginning between age 30 to 35 [12]. Screening and surveillance for less prevalent LS-related cancers may vary depending on family history and should be addressed by a genetic counselling [12].

RRGS should be offered to LS or BRCA carriers who are postmenopausal or not desiring to bear children [14]. For individuals with LS carrying MLH1, MSH2, and MSH6 mutations, the Manchester International Consensus Group recommends risk-reducing total hysterectomy (TH) and bilateral salpingo-oophorectomy (BSO) is offered no earlier than 35–40 years of age [15]. For individuals with LS carrying the PMS2 mutation, the quality of evidence is insufficient to strongly recommend RRGS [15]. Total hysterectomy with BSO (TH-BSO) has been shown to be an effective strategy in individuals with Lynch syndrome, with a 62% and 90% reduction in ovarian and endometrial cancer respectively [16]. As there is insufficient evidence to support TH for BRCA carriers, risk-reducing BSO is recommended between 35 and 40 years of age for BRCA1 carriers and 40–45 years of age for BRCA2 carriers [17]. RRGS may reduce breast cancer incidence by up to 50% and ovarian cancer incidence by 80–96% in individuals with BRCA mutations [17, 18].

Although the reduction in cancer risk is significant for LS and BRCA carriers, deciding whether to undergo RRGS is an emotionally and psychologically challenging task. It is important to weigh the benefits of RRGS against its potential effects on the patient’s physical, mental, and reproductive health. Early surgical menopause is associated with negative outcomes, including increased risk of coronary artery disease, osteoporosis, vasomotor symptoms, sexual dysfunction, and neurocognitive decline [19, 20]. HRT may be an effective intervention in improving effects of premature surgical menopause, including endocrine symptoms, sexual functioning, bone health, and psychological wellbeing [21, 22]. However, HRT use may be associated with increased risk of breast cancer, making surgical menopause an important consideration for patients in the decision-making process to undergo RRGS) [23–25]. Other common concerns contributing to decision-making around RRGS include concerns about body image, risk of heart disease, changes in sexual health, and risk of surgery [25–27]. Preoperative awareness of post-BSO side effects has been found to be highly correlated with patient satisfaction, yet many patients do not have the opportunity to discuss post-BSO functioning with their physicians and seek information independently [28]. To decide whether to undergo RRGS, individuals at risk for gynecologic cancers should be adequately informed about both the benefits and potential effects of prophylactic surgery.

## Methodology

This was a qualitative study approved by the local Hamilton Integrated Research Ethics Board.

### Recruitment

Participants were recruited through the Cancer Genetics Clinic at the Juravinski Cancer Centre, Hamilton, Ontario. Women with suspected genetic syndromes are referred to the Cancer Genetics Clinic for genetic counselling and testing. This study included patients who were offered RRGS with an identifiable BRCA1, BRCA2, and/or LS mutation. Any patient aged 35 to 70 years with a genetic mutation of interest who was offered RRGS was eligible to participate, regardless of when the genetic mutation was identified. Patients who met the inclusion criteria and consented to be contacted for research purposes in the last three years were invited to participate. Participants were asked to review and send back a signed written consent via email prior to the interview.

### Interviews

Interviews were conducted from March 2022 to April 2022 over telephone or virtually on Zoom. Interviews lasted approximately 30 min and used a semi-structured question guide. All participants were prompted to expand upon a core set of topics, including sources of information, concerns regarding surgical side effects, and interactions with healthcare providers during the pandemic. Of 8 participants, 7 consented to being recorded and were transcribed verbatim.

### Data analysis

Thematic analysis was guided by grounded theory. Grounded theory (GT) is a method of inquiry that focuses on creating conceptual frameworks through building inductive analysis. Constant comparative analysis was used to refine ongoing data collection. Themes were discussed by the research team to reach a consensus on the data interpretation.

## Results

Of 17 potential participants, eight completed an interview, seven did not participate due to time constraints, and two could not be reached. All participants were referred for genetic testing due to prior family history of cancer or detection of cancer susceptibility genes in their family. Two women were BRCA2 carriers, five women were diagnosed with Lynch syndrome (three carrying the MSH6 mutations and two carrying the MSH2 mutation), one participant was positive for BRCA2 and the PMS2 mutation associated with LS. The mean participant age was 50 years, with a median of 10 months since completion of genetic testing. Of two patients diagnosed with a BRCA mutation amidst the pandemic, neither had the opportunity to speak with a physician at the time of interview. No participants had undergone RRGS yet. Characteristics of interviewed participants can be found in Table 1.

**Table 1:** Clinical and demographic characteristics of participants

Participant	Age (at time of interview)	# of years prior to interview in which first mutation was identified	Mutation identification	Decision	Cancer history	Menopause
1	38	< 1 year	BRCA2	Yes	No	Premenopausal
2	45	1–3 years	BRCA2 + PMS2	Undecided	No	Premenopausal
3	58	3–5 years	BRCA2	Yes	Yes	Postmenopausal
4	44	< 1 year	MSH2	Undecided (due to not enough information)	No	Premenopausal
5	59	3–5 years	MSH2	Yes	Yes	Postmenopausal
6	42	3–5 years	MSH6	No	No	Premenopausal
7	57	> 5 years	MSH6	Yes	Yes	Postmenopausal
8	56	1–3 years	MSH6	Yes	No	Postmenopausal

Results can be divided into two main themes: (1) patient’s information needs during the decision-making process; (2) the primary sources of information on which patients use to satisfy information needs.

Three key considerations surrounding RRGS were identified: cancer risk, menopause, and psychological readiness (Table 2).

**Table 2:** Key considerations

Key Considerations	Information Needs
Cancer Risk	Explanation of statistical data on risk reduction Resources for patients to review in their own time (e.g. pamphlets, websites)
Menopause	Management of surgical menopause (e.g. hormone replacement therapy, alternative medicine)
Psychological Readiness	Recovery process and duration More time between time of mutation identification and subsequent discussions about RRGS may help women process information Regular conversations about RRGS

### Cancer risk

Perception of cancer risk played the most important role in motivating individuals’ decisions regarding RRGS. Patients with a personal or extensive family history of cancer were more decisive.

*“When you have cancer, you want to do all you can about it.” - Age 58, BRCA2*.

*“It was a no-brainer, especially with family history.” - Age 57, MSH6*.

Quantitative data about risk reduction provided by healthcare professionals were a driving factor in the decision-making process.

*“I want to speak to a doctor and get some medical, statistical data that could be beneficial to my decision-making.” - Age 44, MSH2*.

Some participants found it helpful to use these statistics as a starting point to review resources online in their own time.

*“She says it can develop within 3 months; whereas if you get the surgery, it’s 90% chance you won’t get it, or even higher…I try to remember all these details, but I’m not good with it. I go back online and do my research.” - Age 45, BRCA2, PMS2*.

*“She had a bunch of pamphlets…and gave me the gist. I branched off that and found more information on my own.” - Age 38, BRCA2*.

### Menopause

All postmenopausal women in this study elected to undergo RRGS, with some having previous experience with HRT. Premenopausal women struggled more with weighing the risk of cancer against the effects of surgical menopause and cited age as a barrier to selecting RRGS.

*“If I was older, I would go and get it done. I know I’ll get it done at some point.” - Age 42, MSH6*.

Premenopausal women also expressed the need for more information regarding surgical menopause and interventions to manage side effects.

*“I will definitely be asking my doctor about the early menopause, what it can cause, whether or not I’ll need to do something to prevent heart disease, osteoporosis, all those things that estrogen helps with.” - Age 38, BRCA2*.

### Psychological readiness

The desire to relieve anxiety was a consistent theme amongst women who opted to schedule RRGS. Regular discussions with a healthcare provider helped improve women’s readiness to undergo RRGS.

*“I didn’t want this cloud hanging over me…It was a radical choice. [My doctor] and I have talked about this for over 5 years. It’s something I’m prepared for.” - Age 57, MSH6*.

However, conversations about RRGS may also be uncomfortable and avoided.

*“It’s also scary and I think part of the reason is that I get tested [for endometrial cancer] once a year, so I feel like I can just do that. I don’t really want to have to deal with it, so it’s easier to get caught up in real life.” - Age 45, BRCA2, PMS2*.

### Health care provider

When seeking out information from healthcare professionals, women reported pressure from conversations with their doctors, suggesting the need for more time to process the information and formulate questions about their concerns.

*“I don’t know that I got a good response. I felt like I still had more questions than answers.” - Age 44, MSH2*.

*“My gynecologist said, ‘Alright, we’ll go ahead and book the hysterectomy,’ but I said, no, I still need to talk about it and talk about it because I still had some questions that I needed answered by a doctor and not just by my own research. I felt like he seemed rushed… I was still more hesitant at that point.” - Age 42, MSH6*.

*“My gynecologist is pretty pushy about it. Every time I go, it’s “when are you going to get it done? Get it done, get it done.” - Age 45, BRCA2, PMS2*.

COVID-19 posed a significant obstacle to accessing appointments and engaging in conversations with healthcare providers. Half the women in this study experienced at least a 1 year wait-time between receiving the news about the identification of their mutation from the genetic counsellor and being able to speak with a physician regarding RRGS. Barriers to testing, scheduling, and accessing information induced significant psychological stress.

*“I’ve waited a lot during this pandemic for tests…I understand the strain on the system, but it does play on my mind and affect my ability to sleep. It makes me irritable, it makes me sad. But I can’t change that, I have to accept it.” - Age 59, MSH2*.

*“The stress comes and goes…I think I would have had a surgery booked by now if not for Covid. It was a little disappointing but it is what it is.” - Age 38, BRCA2*.

### Online

When women were not able to have their information needs adequately addressed by their HCP, many used the Internet for information. Women cited websites such as WebMD, Mayo Clinic, and Merck Manuals as some of the sources they consulted.

*“If I hear a term or something, I Google the crap out of it and try to find out as much information as I can.” - Age 38, BRCA2*.

*“I try to keep an open mind and go to what I think are reputable sources. I look for aspects of it, like the type of surgery, what it would involve, how much would my hormones change, things like that.” - Age 45, BRCA2*.

Interactions with HCPs were seen as brief and straight-forward, often without enough time for patients to fully digest the information. The Internet was seen as a method of supplementing or reinforcing what they already knew and was weighed lightly in their decision-making in comparison to information received from HCPs.

*“Anytime I google something I take it with a grain of salt…I feel like all the reliable information I’ve gotten has been through conversations with my doctor. I maybe would understand things a little bit better because it’s explained a little bit easier.” - Age 42, MSH6*.

Some women were part of online support groups in the form of Facebook groups or website forums (e.g. Hysterectomy Sisters, BRCA1 BRCA2 Genetic Ovarian & Breast Cancer Gene). Women used these groups for a variety of information needs as well as for emotional support. Though women were cautious in basing decisions off of information in these groups, some women seemed to benefit psychologically from hearing others’ stories and advice.

*“I had lots of time to [research] because I was waiting [due to Covid]…I belong to a group for Lynch syndrome, and they often share scholarly articles.” - Age 44, MSH2*.

*“There’s a few Facebook groups that I’ve joined that have a lot of information…they talk about the different kinds of surgery and hormone replacement therapy.” - Age 38, BRCA2*.

*“I thought [support groups were] a great resource to hear about other people’s stories and thoughts…but I make my own decisions anyways.” - Age 42, MSH6*.

Some women preferred objective information over subjective experiences and opted to stay out of online support groups to avoid basing information off personal experiences.

*“I just googled Lynch syndrome and looked where it took me. I didn’t look into chat groups, I tried to stick to medical things, and I didn’t want to go into personal experiences…everyone is going to have different experiences and you’ll hear the worst of every experience.” - Age 56, MSH6*.

Particularly within the context of the pandemic, online information may be helpful to relieving the anxiety and discomfort associated with information gaps left by appointment delays.

### Family & friends

All participants in this study were influenced by family and/or friends during their decision-making process. Awareness of family history and second-hand experiences of cancer motivated women to decide to undergo RRGS and helped prepare them mentally for the idea of surgery. Family history of cancer or cancer susceptibility genes also helped women gather information regarding what RRGS entailed.

*“My mom had uterine cancer 20–30 years ago and I watched how rough that was on her, so I’ve been talking to my gynecologist about [getting a hysterectomy] for quite some time, probably 10 years.” - Age 57, MSH6*.

*“I was having conversations ahead of time with family members because they had already gone through [testing]. They had already gotten some information prior to my results coming back and they did some research on Lynch.” - Age 42, MSH6*.

Information from family and friends served primarily as a source of anecdotal evidence about the experience of surgery and its side effects.

*“I just asked them, ‘Is it painful? How did you feel afterwards? How long does it last?” - Age 58, BRCA2*.

However, women acknowledged that information from friends and family would not be able to replace the role of HCPs and expressed the need to have their concerns validated by a physician.

*“I guess some of my information I based off [my friend’s] experience too. She is in a state of menopause and battling with this new, I guess, ‘womanhood’. I guess just having a medical professional walk me through what to expect would have been more helpful, because everybody’s experience can be different.” - Age 44, MSH2*.

## Discussion

This study illustrates the difficulties in decision-making that genetically at-risk women face regarding RRGS, especially amidst the Covid-19 pandemic. Cancer risk and menopause were identified as the most important information needs for patients, with age, cancer history, and family history being the most influential in their decision-making process. In accordance with existing literature, some women did not feel satisfied by their interactions with their physician and reported pressure from their physician to undergo RRGS [29, 30]. Conversations were often viewed as rushed, with a need to further prompt their physician for more information regarding the surgery [31].

Access to care was a significant barrier in women’s decision-making process. Several women expressed concerns regarding delays in booking appointments, which may in large part be due to the COVID-19 pandemic affecting nonessential surgeries and clinic visits. Currently, prophylactic surgeries are listed as a WTIS Priority C by Ontario Health, categorized for patients for whom a delay of 2 months would be unlikely to affect the outcome [32]. However, women in our study reported at least a one-year period between contact by the genetic counsellor and physician, some of whom are still waiting for the chance to speak with a physician regarding the implications of RRGS. Of the women who decided to undergo surgery, none of them have scheduled a date for surgery. These delays result in significant anxiety and psychological stress among women as they wait for test results, counselling, and surgery scheduling.

In situations where patients did not feel adequately informed by their HCP, they frequently consulted outside sources for further information, such online websites and communities, family and friends. Patients primarily used the Internet to educate themselves on aspects of RRGS in preparation for appointments with HCPs amidst Covid-19 delays. Previous literature shows that women may have difficulty processing information during the scheduled appointment time, demonstrating the need for written resources that allow women to process information at their own pace [26]. This points to the potential application of patient decision aids, such as electronic resources and pamphlets, that may be able to more succinctly depict and address patients’ information needs in a visual form. Pilot studies have tested the usability of guided workbooks to help premenopausal BRCA1/2 mutation carriers in their decision-making, but further research may be needed to evaluate the efficacy of these tools [33].

The strengths of our study include a study population of women with LS, BRCA1, and BRCA2 mutations who were diagnosed within the last three years and thus had strong recall of their decision-making process. To our knowledge, this is the first study conducted on women’s information needs regarding prophylactic surgery during the pandemic, as well as the first study on how women evaluate different sources of information to reach a decision.

There are some limitations to this study. Firstly, none of the women interviewed had the opportunity to undergo RRGS and could not comment on themes such as postoperative effects, postoperative care, and recovery needs. Secondly, this study had a small sample size (47% of eligible participants), which may not be representative of the views of the full population. All women who participated were Caucasian.

## Conclusion

In conclusion, this study revealed the key sources of information influencing attitudes regarding RRGS and how women evaluated different sources of information to reach a decision. Results highlight the role of healthcare providers in addressing women’s concerns regarding RRGS, particularly amidst the pandemic. Given the significant psychological stress brought on by the option of RRGS, greater attention to the implications of virtual healthcare should be considered when evaluating patients’ challenges of decision-making during the pandemic. Prioritization of RRGS cases is needed to address significant delays due to the pandemic.

## Data Availability

Not applicable.
